# Dietary Supplementation of Baicalein Affects Gene Expression in Broiler Adipose Tissue During the First Week Post-hatch

**DOI:** 10.3389/fphys.2021.697384

**Published:** 2021-06-25

**Authors:** Yang Xiao, Bailey Halter, Casey Boyer, Mark A. Cline, Dongmin Liu, Elizabeth R. Gilbert

**Affiliations:** ^1^Department of Animal and Poultry Sciences, Virginia Polytechnic Institute and State University, Blacksburg, VA, United States; ^2^Department of Human Nutrition, Foods and Exercise, Virginia Polytechnic Institute and State University, Blacksburg, VA, United States

**Keywords:** flavonoid, baicalein, chicken, adipose tissue, breast muscle

## Abstract

Dietary supplementation of baicalein, a flavonoid, has anti-obesity effects in mammals and broiler chickens. The aim of this study was to determine the effect of dietary baicalein supplementation on broiler growth and adipose tissue and breast muscle deposition. Fifty Hubbard × Cobb-500 day-of-hatch broiler chicks were assigned to a control starter diet or control diet supplemented with 125, 250, or 500 mg/kg baicalein and diets were fed for the first 6 days post-hatch. Body weight, average daily body weight gain, and average daily food intake were all reduced by 500 mg/kg baicalein. Breast muscle and subcutaneous and abdominal fat weights were also reduced in chicks that consumed the baicalein-supplemented diets. mRNAs for genes encoding factors involved in adipogenesis and fat storage, 1-acylglycerol-3-phosphate-O-acyltransferase 2, CCAAT/enhancer-binding protein β, perilipin-1, and sterol regulatory element-binding transcription factor 1, were more highly expressed in the adipose tissue of broilers supplemented with baicalein than the controls, independent of depot. Diacylglycerol acyltransferase and peroxisome proliferator-activated receptor gamma mRNAs, involved in triacylglycerol synthesis and adipogenesis, respectively, were greater in subcutaneous than abdominal fat, which may contribute to differences in expansion rates of these depots. Results demonstrate effects of dietary supplementation of baicalein on growth performance in broilers during the early post-hatch stage and molecular effects in major adipose tissue depots. The mild reduction in food intake coupled to slowed rate of breast muscle and adipose tissue accumulation may serve as a strategy to modulate broiler growth and body composition to prevent metabolic and skeletal disorders later in life.

## Introduction

Excess abdominal fat in broilers, a consequence of selection for growth-related traits, has a negative impact on meat yield and meat quality and contributes to metabolic disorders in the breeders. From 1957 to 2005, there was a greater than 400% increase in the growth rate of broilers (Zuidhof et al., 2014). The 42-day live body weight achieved an increase of 3.3% per year with a 2.55% per year reduction in feed conversion ratio (grams of feed to grams of body weight). Concurrently, breast meat yield increased by 67%, with breast conversion ratio (grams of feed intake converted into grams of breast meat) decreased from 28 to 9.4 at 22–56 days of age (Zuidhof et al., 2014). Liver, which is critical for lipid and carbohydrate metabolism, also increased in its relative weight during the selection to support rapid growth (Zaefarian et al., 2019), as did the abdominal fat content which is positively correlated with breast weight and food intake (Tavárez and Solis de los Santos, 2016). However, in recent years, the demand for white meat has increased due to the lower fat content, and excess adipose tissue, particularly abdominal fat, is a waste product that represents an economic loss to the poultry industry (Yucel and Taskin, 2018). Moreover, in broiler breeders, maintaining optimum body weights and fat percentages are necessitated to ensure optimal reproductive and metabolic health and controlled feeding protocols are initiated at hatch to restrict growth (De Jong and Guémené, 2011). Thus, feed additives that influence appetite and/or body composition are an attractive strategy to modulate growth and fat accretion in broilers and other species.

The use of naturally occurring chemicals in diets has become a popular area of poultry research with the phasing out of antibiotics in broiler production and the search for growth-promoting alternatives. Feed additives already being used in the broiler industry include herbs, spices, and essential oils. Flavonoids, a class of polyphenolic phytochemicals, are among the natural feed additives investigated (Surai, 2014). Baicalein is one such flavonoid that is derived from a root used in traditional Chinese medicine, *Scutellaria baicalensis Georgi*, which has been studied in mammalian and avian species for its effect on obesity and growth performance, respectively.

Adult C57BL/6J mice with high fat diet-induced metabolic disorders had decreased visceral fat weight and no further metabolic exacerbations through dietary supplementation of either *S. baicalensis* or purified baicalein (Pu et al., 2012; Na and Lee, 2019). Daily intraperitoneal administration of baicalin, which is another major compound in *S. baicalensis* root, also decreased visceral fat weight and improved hepatic steatosis in adult Sprague–Dawley rats with high fat diet-induced metabolic disorders (Guo et al., 2009). Consistent with animal studies, an *in vitro* experiment revealed that baicalein suppressed adipogenesis and lipid accretion in 3T3-L1 pre-adipocytes (Seo et al., 2014). We also demonstrated anti-diabetic effects of baicalein, where hyperglycemia, glucose tolerance, and pancreatic islet function were improved in diabetic mice that were fed a baicalein-supplemented diet, without changes in weekly food intake (Fu et al., 2014).

In 42-day-old broilers, dietary supplementation of *S. baicalensis* improved growth performance (Kroliczewska et al., 2008), while dietary baicalein supplementation was associated with reductions in serum total cholesterol, triglycerides and low-density lipoproteins (Zhou et al., 2019). These data all indicate promise in the use of baicalein as a dietary supplement in the poultry industry to support healthy development while mitigating the development of metabolic disorders. Previous studies determined effects at slaughter age, and broilers have a higher average daily body weight gain, lower feed conversion ratio, and more dynamic metabolism at the early post-hatch stage, especially during the first week, when organ maturation is still occurring (Zuidhof et al., 2014). Also, the specific effect of baicalein on fat and lean mass changes as well as corresponding molecular mechanisms are unknown in chickens. Because other studies involving dietary baicalein supplementation focus on later stages of growth and adult animals, the present study sought to examine effects at an earlier age. The objective of this study was thus to investigate the effects of dietary baicalein supplementation on growth performance, breast muscle and adipose tissue development, and mRNA expression of adipogenesis-associated factors in adipose tissue, in broilers during the first 6 days post-hatch.

## Materials and Methods

### Animals

A total of 50 straight run Hubbard × Cobb-500 broiler chicks were obtained on day of hatch from a nearby commercial hatchery. Chicks were individually caged in a room maintained at 30 ± 2°C and 50 ± 5% relative humidity with free access to feed and water and visual and auditory contact with other chicks. Chicks with evenly distributed body weights were assigned to four dietary groups (*n* = 12–13 per group): (1) control diet, a corn and soybean meal-based starter diet formulated to meet recommended requirements for Cobb-500 broilers (Cobb-Vantress; detailed formulation in McConn et al., 2018), (2) low baicalein (125 mg/kg), (3) middle baicalein (250 mg/kg), and (4) high baicalein (500 mg/kg). Dietary baicalein inclusion levels were based on our previous work with mice (Fu et al., 2014). Baicalein (98% pure via HPLC) was purchased from Xi’An Yile Bio-Tech Company, China. Experimental procedures were performed according to the National Research Council Publication and were approved by the Virginia Tech Institutional Animal Care and Use Committee.

### Growth Performance and Organ Weights

Body weight and food intake was recorded for individuals from the time that the chicks arrived in our facility (approximately 5 h after they hatched) and then every 24 h until day 6 (*n* = 12–13 per each of the four groups). Feed conversion ratio was calculated by finding the quotient of food intake and body weight gain. On day 6 post-hatch, all chicks were weighed then euthanized by cervical dislocation. Breast muscle, liver, and subcutaneous (visible adipose tissue connected to skin) and abdominal fat (adipose tissue connected to gizzard) tissues were removed and tissue weights were recorded. Subcutaneous and abdominal fat samples from 10 chicks in each dietary group were transferred to vials containing RNA*later* (Invitrogen, Carlsbad, CA, United States) and stored at −80°C until total RNA isolation. Sex was determined by gonadal inspection postmortem.

### Total RNA Isolation, Reverse Transcription, and Real-Time PCR

Adipose tissue samples were homogenized in TRI Reagent (Sigma-Aldrich, St. Louis, MO, United States) with 5 mm stainless steel beads (Qiagen, Valencia, CA, United States) using a Tissue Lyser II (Qiagen). After the step of addition to 100% molecular biology-grade ethanol, total RNA was purified using the RNeasy Mini kit (Qiagen, CA, United States) according to the manufacturer’s instructions. The eluted total RNA samples were quantified and assessed for purity by spectrophotometry at 260/280/230 nm. First strand cDNA was synthesized from 200 ng total RNA using the High Capacity cDNA Reverse Transcription kit, according to the manufacturer’s instructions (Applied Biosystems, NY, United States). Primers for real-time PCR (Table 1) were designed in Primer Express 3.0 (Applied Biosystems) and validated for amplification efficiency before use (95–105%). A 10 μL reaction contained 5 μL fast SYBR Green Master Mix (Applied Biosystems), 0.25 μL each of 5 μM forward and reverse primers, 3 μL of 10-fold diluted cDNA, and 1.5 μL of nuclease-free water and was duplicated for all samples with an Applied Biosystems 7500 FAST system as described (Zhang et al., 2015).

**TABLE 1:** Primers used for real time PCR^1^.

Gene	Primers sequence (5′-3′); forward/reverse	Accession no.
β*-Actin*	GTCCACCGCAAATGCTTCTAA/TGCGCATTTATGGGTTTTGTT	NM_205518.1
*AGPAT2*	GCCAAACACCGAAGGAACAT/CCATGGCATCCCCAGAGTT	XM_015279793.1
*C/EBP*α	CGCGGCAAATCCAAAAAG/GGCGCACGCGGTACTC	NM_001031459.1
*C/EBP*β	GCCGCCCGCCTTTAAA/CCAAACAGTCCGCCTCGTAA	NM_205253.2
*DGAT2*	TTGGCTTTGCTCCATGCAT/CCCACGTGTTCGAGGAGAA	XM_419374.5
*PLIN1*	GGAGGACGTGGCATGATGAC/GGCCCTTCCATTCTGCAA	NM_001127439.1
*PPAR*γ	CACTGCAGGAACAGAACAAAGAA/TCCACAGAGCGAAACTGACATC	NM_001001460.1
*SREBP1*	CATCCATCAACGACAAGATCGT/CTCAGGATCGCCGACTTGTT	NM_204126.1

*^1^Primers were designed with Primer Express 3.0 (Applied Biosystems). *AGPAT2*, 1-acylglycerol-3-phosphate-O-acyltransferase 2; *C/EBPα*, CCAAT/enhancer-binding protein alpha; *C/EBPβ*, CCAAT/enhancer-binding protein beta; *DGAT2*, diacylglycerol acyltransferase; *PLIN1*, perilipin 1; *PPAR*γ, peroxisome proliferator-activated receptor gamma; *SREBP1*, sterol regulatory element-binding transcription factor 1.*

### Statistical Analysis

The real-time PCR data were analyzed using the ΔΔCT method (Schmittgen and Livak, 2008) with actin as the reference gene and the average of the abdominal adipose tissue from control diet-fed birds as the calibrator sample. The relative quantity (2^–ΔΔ*CT*^) values, growth performance and organ weights were subjected to Fit Model platform in JMP Pro 15 (SAS Ins., Cary, NC, United States). The statistical model for growth performance and organ weights included the main effect of dietary treatment (four doses), time (5 or 6 days) and their interactions. For gene expression, effects included adipose tissue depot (subcutaneous and abdominal), dietary treatment (four doses), and the dietary treatment by adipose depot interaction. Tukey’s test was used *post hoc* for pairwise comparisons and results were considered significant at *P* < 0.05. Sex was excluded from the model as initial tests revealed that no effect involving sex was significant.

## Results

### Growth Performance

**FIGURE 1:**
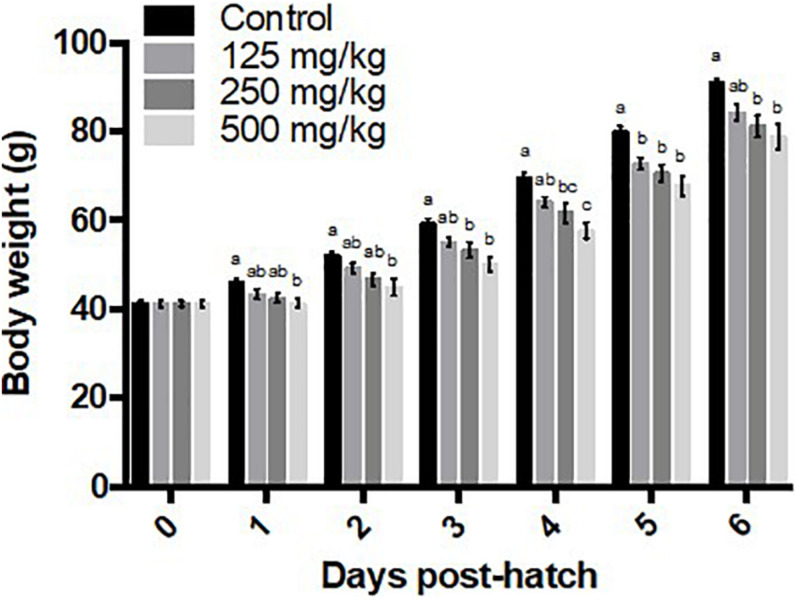
Body weights. Values represent least squares means ± SEM (*n* = 12–13). Different superscripts within each day indicate a significant difference at *P* < 0.05; Tukey’s test.

**FIGURE 2:**
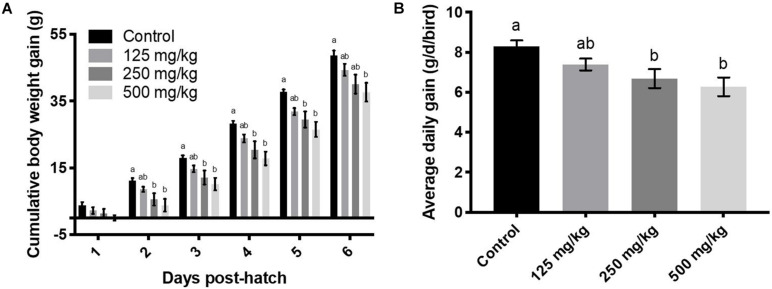
Body weight gain cumulatively **(A)**, and on an average daily basis **(B)**. Values represent least squares means ± SEM (*n* = 12–13). Different superscripts within each day indicate a significant difference at *P* < 0.05; Tukey’s test.

**FIGURE 3:**
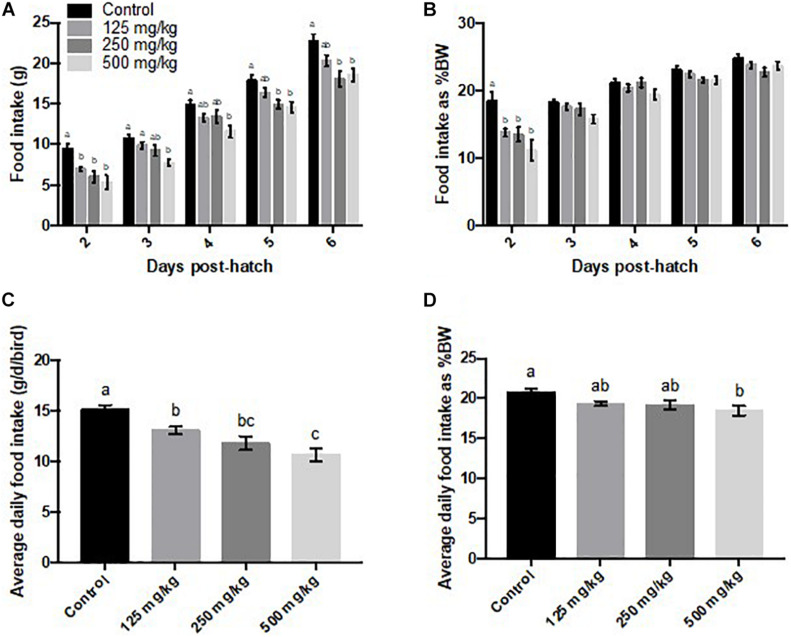
Daily food intake **(A)**, food intake as a percentage of body weight [% body weight (BW)] **(B)**, average daily food intake **(C)**, and average daily food intake as % body weight **(D)**. Values represent least squares means ± SEM (*n* = 12–13). Different superscripts within each day indicate a significant difference at *P* < 0.05; Tukey’s test.

**FIGURE 4:**
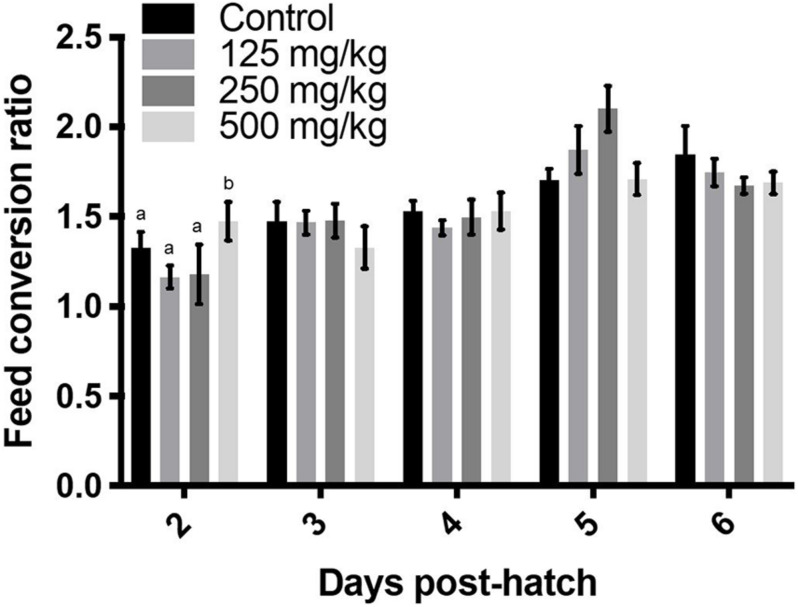
Daily feed conversion ratio over 6 days. Values represent least squares means ± SEM (*n* = 12–13). Different superscripts within each day indicate a significant difference at *P* < 0.05; Tukey’s test.

For results involving more than one time point, differences are depicted graphically for *post hoc* pairwise comparisons at time points for which the main effect was significant (Figures 1–4). The high baicalein (500 mg/kg) diet was associated with a reduction in body weight from days 1 to 6, the middle dose (250 mg/kg) exerted the same effect from days 3 to 6 post-hatch, and the low dose from days 5 to 6, compared to the control group (Figure 1). Both middle and high baicalein doses consistently lowered cumulative body weight gain from days 2 to 5, whereas only the high dose had an effect on day 6, compared to the controls (Figure 2A). Both middle and high baicalein doses lowered the average daily body weight gain over the 6 days of treatment (*P* = 0.003 for main effect of dietary treatment; Figure 2B). Meanwhile, low dose (125 mg/kg) baicalein did not have any effect on body weight or body weight gain.

The high baicalein dose lowered food intake from days 2 to 6 post-hatch, compared to the control group (Figure 3A), while the middle dose affected food intake on days 2, 5, and 6, and the low dose reduced food intake on day 2 post-hatch, relative to chicks that were fed the control diet. When calculated as a percentage of body weight, differences were significant on day 2 post-hatch (Figure 3B). Over the 6 days of observation, average daily food intake was lowered by all levels of baicalein supplementation (*P* < 0.0001; main effect of dietary treatment; Figure 3C), whereas average daily food intake calculated as a percentage of body weight was only lowered in the high baicalein group (*P* = 0.009; main effect of dietary treatment; Figure 3D). Feed conversion ratio was increased in the high baicalein group on day 2 post-hatch (Figure 4). Although the main effect of treatment was also significant for feed conversion ratio at day 5 (*P* = 0.04), no difference was detected among groups using Tukey’s *post hoc* test. The average feed conversion ratio over 6 days of treatment did not differ among the groups (data not shown).

### Organ Weights

Relative abdominal fat weight (as % BW) tended to be reduced by baicalein (*P* = 0.05; main effect of dietary treatment; Table 2), whereas subcutaneous fat was not affected by treatments. Similarly, relative breast muscle weight was also decreased by the high baicalein dose (*P* = 0.005).

**TABLE 2:** The effect of dietary baicalein supplementation on organ weights at day 6 post-hatch in Hubbard × Cobb-500 broilers^1^.

Treatment	Abdominal fat as % BW	Subcutaneous fat as % BW	Liver as % BW	Breast muscle as % BW
0 mg/kg	0.092	0.32	3.92	8.38^*a*^
125 mg/kg	0.092	0.28	4.40	8.02^*a*^
250 mg/kg	0.080	0.21	4.25	7.16^*ab*^
500 mg/kg	0.055	0.23	4.17	6.67^*b*^
SEM	0.01	0.04	0.16	0.34
*P*-value	0.05	0.18	0.25	0.005

*^1^Values represent least squares means and pooled standard errors of the means with associated *P*-values for the effect of dietary treatment (*n* = 10). Different letters within each sampling measurement indicate a significant difference at *P* < 0.05; Tukey’s test.*

### Adipose Tissue Relative mRNA Gene Expression

There was no treatment by adipose tissue depot interaction for any of the genes that were measured. There was a main effect of treatment on the mRNA abundance of 1-acylglycerol-3-phosphate-O-acyltransferase 2 (*AGPAT2*; *P* = 0.01), CCAAT/enhancer-binding protein beta (*C/EBPβ*; *P* = 0.0001), perilipin-1 (*PLIN*; *P* = 0.001), and sterol regulatory element-binding transcription factor 1 (*SREBP1*; *P* = 0.03, Table 3). Expression of *AGPAT2* mRNA was greater in broilers from the middle baicalein dose group than the control or high dose groups. Abundance of *PLIN1* mRNA was greater in the middle dose group than in all other groups, and *SREBP1* was greater in the middle baicalein dose group than in the control group. Expression of *C/EBPβ* mRNA was greater in the high dose baicalein than low or middle dose groups. Independent of treatment, expression of diacylglycerol acyltransferase (*DGAT2*) and peroxisome proliferator-activated receptor gamma (*PPAR*γ) were more highly expressed in subcutaneous than abdominal adipose tissue (*P* = 0.04 and 0.02, respectively).

**TABLE 3:** Effect of baicalein treatment on relative mRNA abundance of adipogenesis-associated factors.

Effect^1^	*AGPAT2*	*C/EBP*α	*C/EBP*β	*DGAT2*	*PLIN1*	*PPAR*γ	*SREBP1*
**Treatment**							
0 mg/kg	1.20^b^	0.93	0.93^ab^	1.52	1.23^b^	1.30	1.18^b^
125 mg/kg	1.75^ab^	2.00	0.52^b^	1.80	2.23^b^	1.21	1.14^ab^
250 mg/kg	3.17^a^	1.28	0.53^b^	1.42	5.27^a^	1.69	2.40^a^
500 mg/kg	1.39^b^	2.13	1.23^a^	2.24	1.86^b^	1.47	1.49^ab^
SEM	0.47	0.48	0.12	0.36	0.79	0.25	0.34
*P*-value	0.01	0.24	0.0001	0.38	0.001	0.53	0.03
**Adipose depot**							
Subcutaneous	1.96	1.11	0.72	2.11	2.84	1.72	1.59
Abdominal	1.80	2.05	0.89	1.38	2.45	1.12	1.51
SEM	0.34	0.34	0.08	0.25	0.56	0.18	0.24
*P*-value	0.74	0.06	0.16	0.04	0.63	0.02	0.83
Treatment × adipose depot	0.25	0.26	0.41	0.66	0.52	0.56	0.42

*^1^Values represent least squares means and pooled standard errors of the means with associated *P*-values for the effect of dietary treatment, adipose tissue depot, and their interaction (*n* = 10). Different superscripts within an effect for each gene are significantly different at *P* < 0.05, Tukey’s test.*

**AGPAT2*, 1-acylglycerol-3-phosphate-O-acyltransferase 2; *C/EBPα*, CCAAT/enhancer-binding protein alpha; *C/EBPβ*, CCAAT/enhancer-binding protein beta; *DGAT2*, diacylglycerol acyltransferase; *PLIN1*, perilipin-1; *PPAR*γ, peroxisome proliferator-activated receptor gamma; *SREBP1*, sterol regulatory element-binding transcription factor 1.*

## Discussion

In this study, we first determined the effect of dietary baicalein supplementation on growth performance and organ weights of broilers during the first week post-hatch. The decrease in body weight and average daily weight gain in response to the middle and high levels of baicalein supplementation was consistent with the observation that 0.5% supplementation of *S. baicalensis* root, which contains 19.3 ± 2.3 mg of baicalein and 214.5 ± 13.3 mg total flavonoids per gram of dry matter, reduced the body weight of 42-day old Hubbard Hi-Y chickens (Króliczewska et al., 2017). In contrast, supplementation of 1.0 and 1.5% of *S. baicalensis* root increased body weight and body weight gain of these birds (Króliczewska et al., 2017), which was similar to what was observed in 42-day old Arbor Acres chickens that were fed diets containing 100 and 200 mg/kg baicalein (Zhou et al., 2019). However, no body weight increase was observed in the same birds at 21 days of age (Zhou et al., 2019).

In the present study, baicalein supplementation was associated with decreased average daily food intake, whereas it was increased in 42-day Hi-Y chickens (Króliczewska et al., 2017) and unchanged in 42-day Arbor Acres broilers (Zhou et al., 2019). Weekly food intake was not affected over the course of 8 weeks of feeding a baicalein-supplemented high-fat diet (500 mg/kg) to middle-aged mice (Fu et al., 2014). Such differences among studies indicate that when interpreting the effect of baicalein on growth performance of broilers, the genetic strains, developmental stage, as well as the form (in flavonoid mixture or purified baicalein), dose, route, and duration of supplementation should all be considered. Also, the results can differ when using an end-point versus day-by-day analysis. The end-point analysis is more reflective of the overall effect at a given time range, whereas it can either bury the significance at a certain time point (like the non-significant average feed conversion ratio versus the significance on day 2 post-hatch in Figure 4), or reveal an overall significance by distributing the significance on 1 day to the other days that lack significance (for instance, the daily versus average food intake as percentage of body weight in Figure 3). It is worth noting that the two experiments conducted by the same group, using the same strains of birds at the same age, identified different effects of the same doses of *S. baicalensis* root supplementation on body weight, feed conversion ratio, body weight gain, as well as food intake (Kroliczewska et al., 2008; Króliczewska et al., 2017). The disparate effects indicate that boilers may have different sensitivities to flavonoid supplementations over years of selection for rapid growth. It is also possible that the root extracts contain varying amounts of bioactive chemicals that contribute to the biological effects observed, whereas results are expected to be more robust when using a pure chemical.

To our knowledge, there are no reports concerning the effect of baicalein or *S. baicalensis* on body fat mass changes or the corresponding molecular mechanisms, in an avian model. Our results demonstrated that baicalein supplementation reduced absolute abdominal and subcutaneous fat masses (data not shown) and the relative abdominal fat mass in broilers during the early post-hatch stage. Adult C57BL/6J mice with metabolic disorders induced by chronic high fat diet consumption had decreased visceral fat weight (including epididymal, mesentery, and abdominal adipose tissue depots) after 29 weeks of 400 mg/kg baicalein supplementation (Pu et al., 2012). Similarly, adult C57BL/6J mice with high fat diet-induced insulin resistance had reduced epididymal fat weight when supplemented with 500 mg/kg body weight of *S. baicalensis* for 9 weeks (Na and Lee, 2019). Similar to the results of dietary baicalein supplementation, Sprague-Dawley rats with high fat-induced metabolic disorders also showed decreased total visceral (including perirenal and epididymal adipose depots) and epididymal fat masses after 16 weeks of daily 80 mg/kg/d intraperitoneally administered baicalin (baicalein is the aglycone form of baicalin) (Guo et al., 2009). However, in these studies, there was either the lack of a control group fed a normal diet with baicalin/*S. baicalensis* administration/supplementation (Guo et al., 2009; Na and Lee, 2019), or such group showed no difference in adipose tissue depot weights compared to those fed a normal diet that did not contain baicalein (Pu et al., 2012).

To begin to elucidate the molecular mechanisms involved in adipose tissue weight changes during dietary baicalein supplementation, we next determined expression of major genes involved in adipogenesis. Although both abdominal and subcutaneous fat weights were reduced by baicalein treatment, Tukey’s test detected pairwise differences only in the abdominal fat depot. Expression of the master regulator of adipogenesis, *PPAR*γ (Tontonoz et al., 1994) and the gene encoding the key enzyme in triglyceride biosynthesis, *DGAT2* (Cases et al., 2001), were greater in the subcutaneous than abdominal depot. This is consistent with our previous finding in low- and high-body weight-selected chickens, where during the early post-hatch stage, adipogenesis is more dynamic in the subcutaneous than abdominal adipose tissue depot (Xiao et al., 2019). Possibly, reduced adipogenesis in the abdominal depot contributed to its reduced expansion in chicks fed diets supplemented with baicalein. Interestingly, although the depot weight reduction was only significant at a high level of dietary supplementation, *AGPAT2*, *PLIN1*, and *SREBP1* were more highly expressed in the middle dose of baicalein than control diet group, with levels in the high dose group comparable to the controls. *C/EBPβ* is a transcription factor that is activated during the early stage of adipogenesis, and further activates *C/EBPα* and *PPAR*γ (Tang and Lane, 2012). In differentiated 3T3-L1 cells, mRNA expression of *C/EBPβ* and protein expression of C/EBP*α*, PPAR*γ*, SREBP1, and DGAT1, which are involved in adipogenesis and lipid accumulation, were all decreased by baicalein treatment in a dose-dependent manner (3.125, 6.25, and 12.5 μ*M*) at 6 days post-treatment, which was also confirmed by an *in vivo* study using zebrafish (Seo et al., 2014). Although relative lipid accumulation was also reduced in a dose-dependent manner, when cells were treated with high dose baicalein during differentiation, a significant reduction in lipid accumulation was only observed from day 2 post-differentiation (Seo et al., 2014). Another *in vitro* study using a single dose of 50 μ*M* baicalein with 3T3-L1 cells also demonstrated decreased *C/EBPα*, *PPARγ*, and *SREBP1* expression on day 6 post-differentiation, whereas lipid content was only decreased during the first 2 days post-differentiation, but not the remaining 4 days, although the overall lipid content over 6 days was also lower than in the control group (Nakao et al., 2016).

These results suggest that adipocytes at various differentiation stages have different sensitivities to baicalein treatment. It is possible that during early development post-hatch, cells in adipose tissue display high rates of hyperplasia (increase in cell number) and hypertrophy (increase in cell volume), whereas at later developmental stages of life, since adipocyte precursor cells are not as adipogenic, hypertrophy predominates as the major contributor to lipid deposition and adipose tissue expansion (Xiao et al., 2019), which together result in a differential sensitivity to baicalein treatment. Another critical factor pertinent to the present study is that chicks utilize residual yolk as an energy source. Yolk, which contains over 20% of lipids, is essentially absorbed around 4 days post-hatch (Moran, 2007). No research has been conducted on the effect of baicalein on yolk sac resorption in chicks. It is possible that baicalein also interferes with yolk utilization and thereby impacts lipid metabolism in adipose tissue depots, although these data were not collected and beyond the scope of the present study.

In contrast to the findings that 0.5 and 1.5% baical skullcap root supplementation increased dry matter percentage of the breast muscles in Hi-Y broilers at 42 days of age (Kroliczewska et al., 2008), we observed a decrease in absolute and relative breast muscle weight in 7-day old Hubbard × Cobb-500 chicks that consumed a diet containing 500 mg/kg baicalein. The continuous selection for high carcass yield has resulted in an over 80% increase in pectoralis major from 1957 to 2005 (Zuidhof et al., 2014). However, the intense selection also led to an unbalanced body composition, which can cause a plethora of disorders, such as “water belly” (because of the insufficient oxygen supply by relatively small heart and lungs and excessive growth) and “green muscle disease” (also because of the insufficient oxygen supply-induced ischemia and sequential necrosis) (Bailey et al., 2015; Soglia et al., 2019). Therefore, a mild attenuation of breast muscle weight gain through baicalein supplementation may indicate a protective effect of baicalein during early post-hatch broiler development. By slowing down the expansion of breast muscle, the chance for rapid growth-induced body distortion may be prevented as is the sequential induction of the above-mentioned diseases. It is also possible that there is a catch-up growth in response to baicalein supplementation at a later stage of life. Extended feeding experiments should be conducted to illustrate if such a reduction in yield is persistent during later development or if there is compensatory growth leading to comparable or even higher meat yield at 42 days of age.

Our study thus extended the previous research on the effects of baicalein on growth performance in broilers to the early post-hatch stage and for the first time elucidated its effects on major adipose tissue depots as well as associated molecular mechanisms. Such information provides insights on its possible use as a dietary supplement in the poultry industry during early development to modulate rapid growth, slow the accretion of abdominal fat, and to induce a mild reduction in feed intake that circumvents the need to employ a feed restriction protocol early in life. In conclusion, dietary supplementation of baicalein during the early rearing phase may have multi-faceted benefits that improve broiler welfare, health, and overall productivity. For recommended supplementation levels, it is anticipated that further research will reveal the minimum dietary inclusion amount that yields the expected benefits most economically. The present results suggest that the middle dose modulated performance modestly.

## Data Availability Statement

The raw data supporting the conclusions of this article will be made available by the authors, without undue reservation.

## Ethics Statement

The animal study was reviewed and approved by the Virginia Tech Institutional Animal Care and Use Committee, Virginia Tech, Blacksburg, VA, United States.

## Author Contributions

MC and EG contributed to conception and design of the research, interpretation of results, and editing of the manuscript. CB performed the experiments, contributed to statistical analysis, and interpretation of results. YX contributed to statistical analysis, interpretation of results, and drafted the manuscript. BH contributed to interpretation of results and wrote sections of the manuscript. DL contributed to design of the research and interpretation of the results. All authors contributed to manuscript revision, read, and approved the submitted version.

## Conflict of Interest

The authors declare that the research was conducted in the absence of any commercial or financial relationships that could be construed as a potential conflict of interest.
